# The diagnostic utility of urinary 5-HIAA and leucine-rich alpha-2 glycoprotein in acute appendicitis: a narrative review

**DOI:** 10.3389/fmed.2025.1605160

**Published:** 2025-06-06

**Authors:** Yahiya Baig, Aamer Mohammed, Alexandra E. Butler

**Affiliations:** ^1^School of Medicine, Royal College of Surgeons in Ireland-Medical University of Bahrain, Busaiteen, Bahrain; ^2^Research Department, Royal College of Surgeons in Ireland-Medical University of Bahrain, Busaiteen, Bahrain

**Keywords:** acute appendicitis (AA), urinary biomarkers, bacteriuria, 5-Hydroxyindoleacetic acid (5-HIAA), leucine-rich alpha-2 glycoprotein (LRG), urinary tract infection (UTI)

## Abstract

**Background:**

Acute appendicitis (AA) remains diagnostically challenging despite its global prevalence. Current methods rely on clinical scoring systems (e.g., Alvarado score) and imaging (US, CT, and MRI). Urinary biomarkers like 5-hydroxyindoleacetic acid (5-HIAA) and leucine-rich alpha-2 glycoprotein (LRG) offer non-invasive potential, reflecting intestinal inflammation and neutrophilic activity, respectively. This review evaluates their diagnostic accuracy.

**Methods:**

A targeted literature review was conducted using PubMed, Scopus, and ScienceDirect (2004–April 2025) to identify studies investigating urinary 5-HIAA and LRG in AA. Inclusion criteria focused on peer-reviewed studies reporting diagnostic accuracy, biomarker performance, and clinical utility. Data were extracted from 13 studies (2,623 participants) for 5-HIAA and 11 studies (1,586 participants) for LRG, including meta-analyses where available. Results were synthesized narratively, with emphasis on sensitivity, specificity, and area under the curve (AUC) metrics.

**Results:**

5-HIAA demonstrated variable diagnostic performance, with pooled sensitivity of 68.6% and specificity of 82% (AUC ~0.64). While it showed higher sensitivity (82%) in perforated appendicitis, its utility in uncomplicated cases was limited by dietary interference and methodological heterogeneity. In contrast, LRG exhibited greater consistency, particularly in pediatric populations. Serum LRG achieved an AUC of 0.95, while creatinine-adjusted urinary LRG, when combined with clinical variables [e.g., appendicitis urine biomarker (AuB) score], reached 97.6% sensitivity for ruling out AA. However, standalone urinary LRG had low sensitivity (17.65%), highlighting its role as an adjunct rather than an independent diagnostic tool. Both biomarkers performed optimally when integrated with clinical scoring systems (e.g., pediatric appendicitis score) or imaging.

**Conclusions:**

While 5-HIAA and LRG offer non-invasive diagnostic potential, neither biomarker is sufficient as a standalone test for AA. 5-HIAA may aid in perforation risk stratification, whereas LRG excels in ruling out AA, particularly in pediatric cases. Future research should focus on standardizing assays, validating multimodal biomarker panels [e.g., 5-HIAA + LRG + CRP (C-reactive protein)], and developing point-of-care applications to enhance clinical feasibility. Until then, these biomarkers should complement—not replace—existing diagnostic strategies, serving as valuable adjuncts in ambiguous or high-risk presentations.

## 1 Introduction

**Figure 1:**
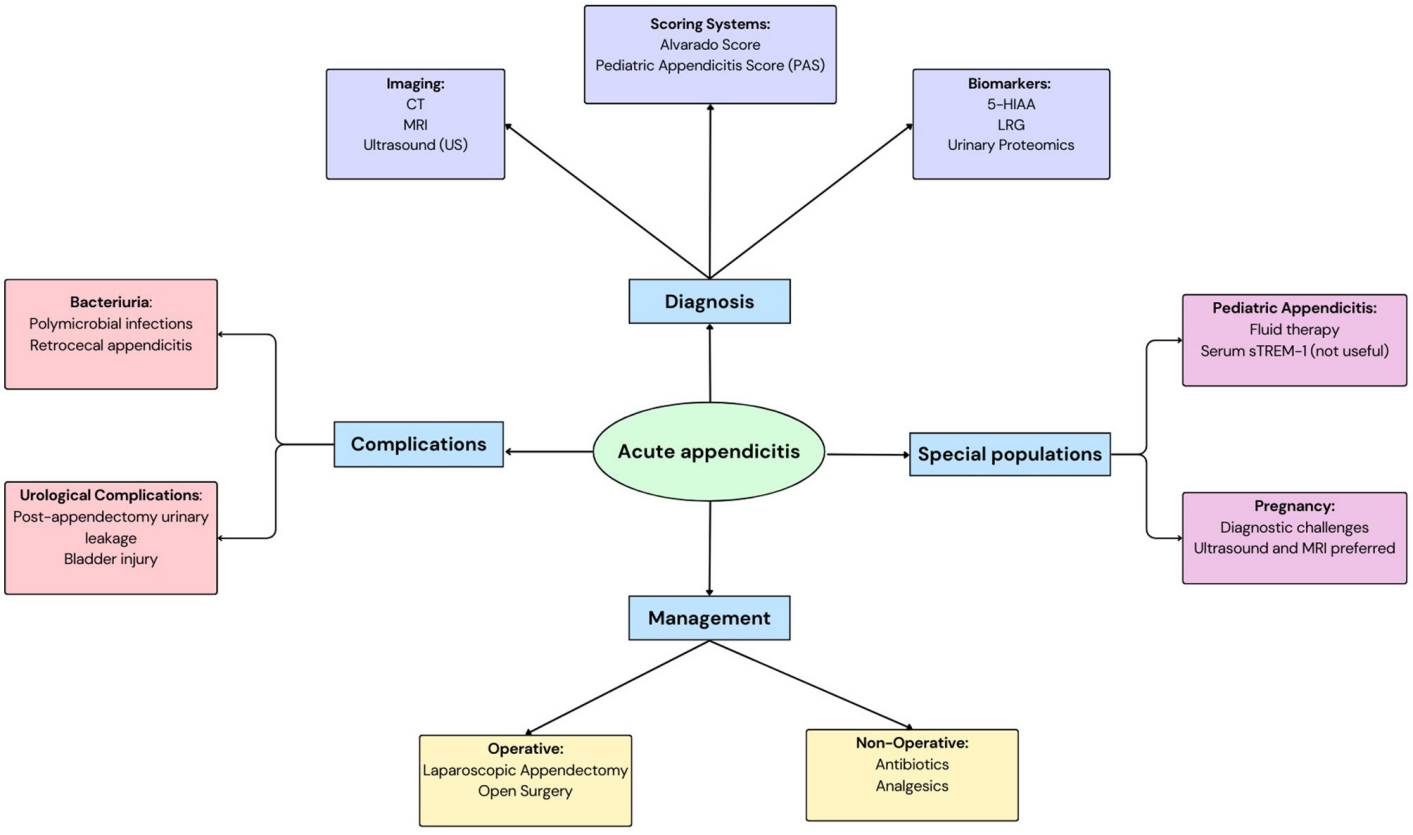
A schematic to depict acute appendicitis: its diagnosis, management, complications and considerations in special populations.

Acute appendicitis (AA) is a common surgical emergency with peak incidence occurring between ages 10 and 30 years and a well-established male predominance (1, 2). Complicated appendicitis upon presentation occurs in 16%−40% of cases, with higher rates observed in both pediatric populations and patients over 50 years (3). Despite advances in diagnostic imaging and laboratory testing, misdiagnosis remains a challenge, leading to negative appendectomy rates ranging from 0 to 46% (4). Clinical evaluation remains challenging due to overlapping symptoms with other abdominal pathologies, variability in presentation (e.g., pediatric, geriatric, or atypical cases), and limitations of existing biomarkers (e.g., CRP, WCC). Two key biomarkers-−5-hydroxyindoleacetic acid (5-HIAA) and leucine-rich alpha-2 glycoprotein (LRG), have emerged as non-invasive diagnostic candidates. 5-HIAA, the primary metabolite of serotonin, reflects intestinal enterochromaffin cell activity and systemic inflammation, with elevated urinary levels reported in appendiceal perforation due to local serotonin release (5, 6). LRG, an acute-phase glycoprotein, is upregulated in neutrophilic inflammation and has demonstrated utility in differentiating complicated appendicitis in pediatric cohorts (7, 8). This review evaluates the diagnostic performance, clinical utility, and limitations of these biomarkers in AA, focusing on their integration with existing diagnostic strategies (7, 8) (Figure 1).

Several scoring systems, including the Alvarado score and pediatric appendicitis score (PAS), aid in diagnosing appendicitis, particularly in pediatric populations. The Alvarado score (range: 1–10) categorizes scores of 1–4 as negative, 5–8 as indeterminate (requiring further evaluation), and 9–10 as diagnostic, a combined sensitivity of 76.0% and a combined specificity of 71.0% (9). The PAS (range: 1–10) classifies scores of 1–3 as negative, 4–7 as indeterminate, and 8–10 as positive, with 70.9%−98.7% sensitivity and 91.5%−95.7% specificity (10, 11). Given their moderate accuracy and reliance on subjective clinical findings (e.g., rebound tenderness, anorexia), these scores are best used to triage patients for imaging or biomarker testing, rather than as standalone diagnostics.

## 2 Methodology

A comprehensive literature search was conducted using PubMed, Scopus, ScienceDirect to identify relevant peer-reviewed studies published in English between 2004 and April 2025. Additional references were screened from the bibliographies of selected articles. The search strategy combined keywords related to AA with terms related to urinary biomarkers and bacteriuria, specifically: (“acute appendicitis” OR “appendicitis”) AND (“5-HIAA” OR “5-Hydroxyindoleacetic Acid” OR “leucine-rich alpha-2 glycoprotein” OR “leucine” OR “LRG”) AND (“urine” OR “urinary”). Studies were included if they investigated the role of urinary biomarkers or bacteriuria in the diagnosis, management or differentiation of AA, reported associations between urinary findings and clinical outcomes, and were published in peer-reviewed journals in English. Studies were excluded if they did not examine 5-HIAA or LRG in AA, focused on other abdominal conditions without relevance to AA, had limited scientific relevance (e.g., case reports, non-peer-reviewed abstracts), or were not published in English.

**Figure 2:**
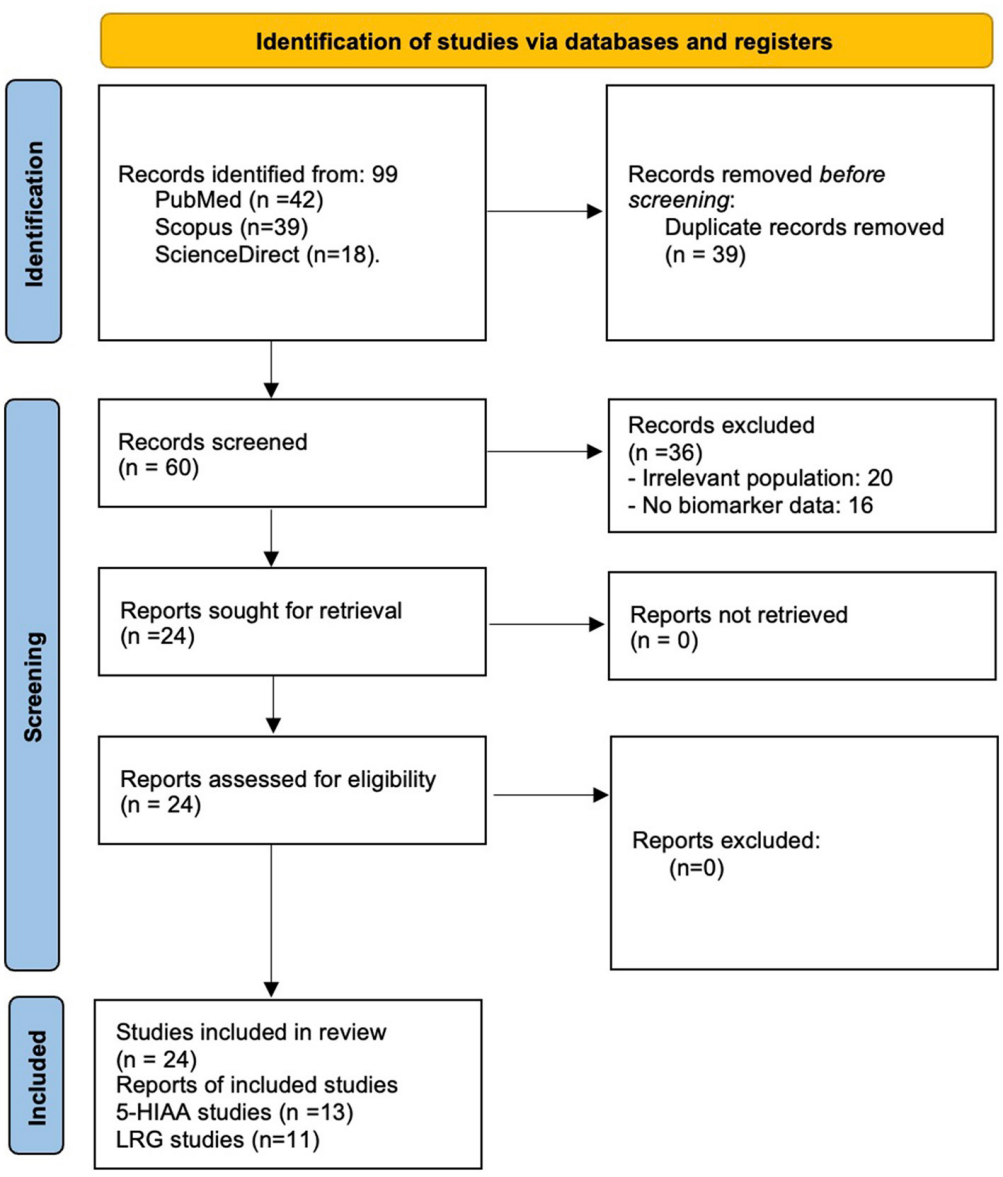
A PRISMA flowchart of selection process for studies included in this literature review.

Following PRISMA guidelines for transparent reporting, our systematic search identified 99 records from PubMed (*n* = 42), Scopus (*n* = 39), ScienceDirect (*n* = 18). After removing 39 duplicates, we screened 60 unique records by title/abstract. Of these, 36 records were excluded at screening stage for not meeting inclusion criteria. Twenty-four full-text articles were assessed for eligibility, ultimately being included (5-HIAA: *n* = 13; LRG: *n* = 11) in the final analysis. The selection process is summarized in Figure 2. The methodological quality of included studies was assessed using the QUADAS-2 tool, evaluating four domains: patient selection, index test, reference standard, and flow/timing. Risk of bias was categorized as low, moderate, or high based on study design, blinding, and adherence to standardized protocols. A total of 2,623 participants from 13 studies, along with a systematic review of 62 studies (participant data not reported) investigating urinary 5-HIAA in AA were included, and total of 1,586 participants from 11 studies investigated LRG in AA were included. Data were extracted independently by two authors, with discrepancies resolved through consensus. Extracted data included study design (e.g., prospective, retrospective, and case-control), population characteristics (e.g., age, sex), urinary biomarkers analyzed (e.g., 5-HIAA, LRG), methods of biomarker measurement (e.g., ELISA, mass spectrometry), urinalysis findings (e.g., hematuria, pyuria, and bacteriuria), and clinical outcomes (e.g., diagnostic accuracy, differentiation of AA from other conditions, prediction of complications). Studies were grouped according to the urinary biomarkers investigated, results were synthesized narratively, grouped by biomarker and clinical application.

## 3 Role of urinary biomarkers in diagnosing acute appendicitis

### 3.1 5-hydroxyindoleacetic acid (5-HIAA)

Haji Maghsoudi et al. (12) included 129 patients with right iliac fossa pain, of whom 96 underwent appendectomy. Among these, 81 were diagnosed with AA, while 15 had a negative appendectomy. The mean urinary 5-HIAA levels were 7.27 μmol/L in the AA group and 9.27 μmol/L in the negative appendectomy group, with no statistically significant difference. Using a cutoff value of 7.4 μmol/L, the sensitivity, positive predictive value (PPV), and negative predictive value (NPV) of the 5-HIAA test were 54.3%, 27.1%, and 55.7%, respectively. The authors concluded that urinary 5-HIAA is not a reliable diagnostic tool for AA due to its poor predictive ability (Table 1) (12).

**Table 1:** Diagnostic performance of urinary 5-HIAA in acute appendicitis.

**Study**	**Population**	**Biomarker**	**Cutoff**	**Sensitivity (%)**	**Specificity (%)**	**AUC**	**Notes**
Haji Maghsoudi, Soltanian (12)	129 patients (81 confirmed appendicitis, 48 non-appendicitis)	Urinary 5-HIAA	7.4 μmol/L	54.3	72.9	NR	Low sensitivity; not superior to routine diagnostics
Mohammadi Tofigh, Samsami (13)	150 patients (40 perforated appendicitis, 110 non-perforated)	Urinary 5-HIAA	Not explicitly stated (mean: 0.5 vs. 0.3 mg/dl)	82	62	NR	Higher 5-HIAA in perforated appendicitis (0.5 ± 0.03 mg/dl) vs. non-perforated (0.3 ± 0.04 mg/dl; *p* < 0.001)
Khirallah and Abdel Ghafar (14)	191 children (3–18 years) with suspected appendicitis	Urinary 5-HIAA	>15 mg/g creatinine	91.8	87.1	0.923	Combined with PAS: AUC improved to 0.958 (93.4% sensitivity, 90.1% specificity)
Arredondo Montero, Bueso Asfura (15)	Meta-analysis (*n* = 1,467)	Urinary 5-HIAA	7.4–27.20 μmol/L (range)	68.6 (pooled)	82.0 (pooled)	NR	High heterogeneity
Bosak Versic, Glavan (16)	93 children (81 AA, 12 non-AA) + 102 controls	Urinary 5-HIAA (HPLC)	Median: 21.18 μmol/L	60.4	48.9	0.55 (0.47–0.62)	No significant difference between AA/non-AA/controls (*p* = 0.48)
Jangjoo, Varasteh (17)	70 patients with suspected appendicitis	Urinary 5-HIAA	5.25 mg/L	44	81	NR	Low sensitivity; useful in advanced appendicitis but not early stages
Rao, Wilson (18)	97 patients (38 AA, 59 non-AA)	Urinary 5-HIAA	19 μmol/L	71	50	0.64	Poor diagnostic accuracy; no correlation with severity. Outperformed by CRP (AUC: 0.76)
Bolandparvaz, Vasei (19)	110 patients (39 AA, 21 GA, 50 controls)	Urinary 5-HIAA (HPLC)	10 μmol/L	84	88	0.90	High diagnostic accuracy for early AA; levels decrease in gangrenous cases. Outperforms CRP/WBC
Hernandez, Jain (20)	100 adults (64 AA: 52 acute, 12 gangrenous/perforated)	Urinary 5-HIAA (HPLC)	10 μmol/L	63 (50.4–6.5)	33 (20–46.7)^*^	NR	No significant difference between acute vs. gangrenous/perforated AA
			20 μmol/L	40 (27–53.7)^*^	63 (48.8–76.2)^*^	NR	
Acharya, Markar (21)	62 studies (systematic review)	Urinary 5-HIAA	NR	72 (68–76)	86 (80–92)	0.88	High specificity but slow processing; cost £21. Best AUC among biomarkers
Ilkhanizadeh, Owji (22)	166 patients (66 AA), 40 controls	Urinary 5-HIAA (HPLC)	20 μmol/L	98	100	NR	Exceptional diagnostic accuracy in suspected cases, however NPV 93% suggests strong rule-out capability
Mentes, Eryilmaz (23)	35 rabbits (21 with appendicitis)	Urinary 5-HIAA (HPLC)	4.15 mg/g creatinine	85	64	0.805	Levels peak in early appendicitis (12 h) and decreases in gangrenous/late-stage cases
Xu, Zhang (24)	15 AA patients, 15 controls	Urinary 5-HIAA (HPLC-ED)	20 μmol/L	NR	NR	NR	Found 4 × higher 5-HIAA in AA patients

5-HIAA, 5-hydroxyindoleacetic acid; PAS, pediatric appendicitis score; AuB, appendicitis urine biomarker score; PAA, pediatric acute appendicitis; GA, gangrenous appendicitis; MD, mean difference; NR, not reported; AUC, area under the curve; CT, computed tomography; US, ultrasonography; ROC, receiver operating characteristic; PPV, positive predictive value; NPV, negative predictive value.

Mohammadi Tofigh et al. (13) conducted a prospective study to investigate the utility of 5-HIAA levels in differentiating perforated appendicitis from non-complicated AA. The study included 150 patients, divided into 40 with perforated appendicitis and 110 with non-complicated appendicitis, all of whom underwent appendectomy with pathological confirmation. Urine spot samples were collected preoperatively, and 5-HIAA levels were measured using ELISA, with samples preserved using hydrochloric acid to maintain stability. The results showed that 5-HIAA levels were significantly higher in the perforated appendicitis group (0.5 ± 0.03 mg/dl) compared to the non-complicated group (0.3 ± 0.04 mg/dl; *p* < 0.001). The sensitivity, specificity, positive predictive value (PPV), negative predictive value (NPV) and diagnostic accuracy of 5-HIAA for predicting perforation were 82%, 62%, 75%, 77%, and 88%, respectively. The authors concluded that 5-HIAA levels could serve as a potential biomarker to identify perforated appendicitis, aiding in surgical planning and decision-making. However, they acknowledged limitations such as the small sample size, influenced by the COVID-19 pandemic and resource constraints, as well as the potential confounding effects of dietary and pharmacological factors on 5-HIAA levels. These results highlight 5-HIAA's potential as a non-invasive tool for assessing perforation severity, though further research is needed to validate its clinical utility (Table 1) (13).

The randomized controlled trial by Khirallah and Abdel Ghafar (14) investigated the diagnostic utility of urinary 5-Hydroxyindoleacetic Acid (5-HIAA) in children with suspected AA. The study included 191 children randomized into two groups: one evaluated using the standard protocol [pediatric appendicitis score (PAS), C-reactive protein (CRP) and ultrasound] and the other with the addition of urinary 5-HIAA measurement. The results demonstrated that urinary 5-HIAA had an area under the curve (AUC) of 0.923, with a sensitivity of 91.8% and specificity of 87.1% at a cutoff of >15 mg/g creatinine. When combined with PAS, the diagnostic accuracy improved further, with an AUC of 0.958, sensitivity of 93.4%, and specificity of 90.1%. The study also noted a significant reduction in negative appendectomy rates and readmissions in the group where 5-HIAA was used. However, urinary 5-HIAA levels did not significantly differ between histopathological types of appendicitis (catarrhal, suppurative, perforated, or gangrenous), though levels tended to increase with disease severity. These findings suggest that urinary 5-HIAA is a promising non-invasive biomarker for diagnosing AA in children, particularly when combined with clinical scoring systems like PAS. However, its inability to distinguish between complicated and uncomplicated appendicitis limits its utility in predicting disease severity. This study supports 5-HIAA's role as a diagnostic adjunct in pediatric AA, particularly when combined with PAS (Table 1) (14).

The systematic review by Arredondo Montero et al. (15) provides valuable insights into the diagnostic performance of urinary 5-HIAA in AA, synthesizing data from 12 studies involving 1,467 participants. The meta-analysis revealed that urinary 5-HIAA levels were significantly higher in AA patients compared to controls, with a pooled sensitivity of 68.6% and specificity of 82%. However, the review highlighted significant heterogeneity among studies, particularly due to variations in control groups (e.g., healthy individuals, negative appendectomies, and non-surgical abdominal pain) and measurement techniques [e.g., high performance liquid chromatography (HPLC) vs. enzyme linked immunosorbent assay (ELISA)]. While urinary 5-HIAA shows promise as a non-invasive diagnostic tool, its utility is limited by factors such as dietary, pharmacological, and medical conditions that can elevate 5-HIAA levels. Additionally, the review found that 5-HIAA is not useful for distinguishing between non-complicated (NCAA) and complicated appendicitis (CAA). These findings underscore the need for further research to validate urinary 5-HIAA as a reliable biomarker and explore its integration with other urinary biomarkers (e.g., LRG, proteomics) and bacteriuria in the diagnostic framework for AA (15). These findings highlight urinary 5-HIAA's limited but potential role in AA diagnosis, emphasizing the need for standardized methodologies and multimodal biomarker approaches to enhance diagnostic precision (Table 1).

Bosak Versic et al. (16) prospectively evaluated urinary 5-HIAA in 93 children with suspected appendicitis (81 confirmed AA, 12 non-AA) and 102 healthy controls using HPLC. Contrary to the hypothesis that 5-HIAA—a serotonin metabolite released during appendiceal inflammation—would be elevated in AA, the study found no significant difference in median 5-HIAA levels between AA (22.97 μmol/L), non-AA (22.52 μmol/L), and control groups (24.68 μmol/L; *p* = 0.48). ROC analysis demonstrated poor diagnostic accuracy (AUC: 0.55, 95% CI: 0.47–0.62), with low sensitivity (60.4%) and specificity (48.9%). Even when adjusted for creatinine, 5-HIAA failed to differentiate AA severity (phlegmonous/gangrenous/perforated) or distinguish AA from other abdominal pain etiologies. The authors concluded that spot urinary 5-HIAA is unreliable for diagnosing pediatric AA, aligning with prior inconsistent adult studies but contradicting earlier pediatric findings [e.g., (14)]. Key limitations included small sample size and lack of dietary controls, though the study remains the largest pediatric investigation of 5-HIAA to date. This study challenges the utility of urinary 5-HIAA in pediatric AA, underscoring its poor diagnostic accuracy and the necessity for alternative biomarkers in this population (Table 1) (16).

Jangjoo et al. (17) investigates the diagnostic value of urinary 5-hydroxyindoleacetic acid (U-5-HIAA) in AA, comparing its performance with traditional markers like leukocyte count and neutrophil percentage. The authors conducted a double-blind study on 70 patients, measuring U-5-HIAA via ELISA and analyzing its correlation with histopathological findings. The results showed that U-5-HIAA had low sensitivity (44%) but high specificity (81%) at a cutoff of 5.25 mg/L, making it insufficient as a standalone diagnostic test. Notably, U-5-HIAA levels were significantly higher in advanced appendicitis (e.g., gangrenous or perforated cases) but not in early-stage disease. The study concludes that while U-5-HIAA may aid in diagnosing severe appendicitis, it cannot replace clinical assessment or imaging. While urinary 5-HIAA may assist in identifying advanced appendicitis, its low sensitivity limits its clinical utility, warranting further investigation into complementary diagnostic tools (Table 1) (17).

Rao et al. (18) prospectively evaluated spot urinary 5-HIAA in 97 patients with suspected appendicitis, comparing its diagnostic performance to conventional biomarkers (WCC, CRP, and neutrophil count) and Alvarado scores. While median 5-HIAA levels were higher in confirmed appendicitis cases (24.19 vs. 18.87 μmol/L; *p* = 0.038), the test demonstrated poor discriminative ability, with low sensitivity (71%) and specificity (50%) at a 19 μmol/L cutoff. ROC analysis revealed inferior diagnostic accuracy (AUC: 0.64) compared to CRP (AUC: 0.76) and other blood markers. Notably, 5-HIAA levels did not correlate with appendicitis severity (*p* = 0.704), and its addition to biomarker panels did not significantly improve AUC (0.77 vs. 0.76 without 5-HIAA). The authors concluded that urinary 5-HIAA lacks clinical utility for diagnosing AA, aligning with prior studies highlighting its inconsistent performance (Table 1) (18).

Bolandparvaz et al. (19) investigated urinary 5-HIAA (U-5-HIAA) as a diagnostic biomarker for AA in 110 patients, comparing it with CRP, WBC, and neutrophil counts. Using HPLC, they found significantly elevated U-5-HIAA levels in non-gangrenous AA (32 ± 2.6 μmol/L) compared to controls (4.1 ± 0.5 μmol/L, *p* < 0.001), with gangrenous appendicitis (GA) showing intermediate levels (13.8 ± 2.1 μmol/L). At a cutoff of 10 μmol/L, U-5-HIAA demonstrated 84% sensitivity and 88% specificity, outperforming CRP (AUC: 0.66) and WBC (AUC: 0.70) with an AUC of 0.90. Notably, U-5-HIAA levels declined in GA, suggesting reduced serotonin secretion due to tissue necrosis. The study concluded that spot U-5-HIAA is a superior diagnostic marker for early AA but loses predictive value in advanced disease, highlighting its potential for early detection while underscoring limitations in perforated cases (Table 1) (19).

Hernandez et al. (20) prospectively evaluated urinary 5-HIAA in 100 adults (aged 18–70 years) with suspected appendicitis using HPLC. Of 72 appendectomies, 64 had confirmed appendicitis (52 acute, 12 gangrenous/perforated). The study found no significant diagnostic utility for 5-HIAA: mean levels were similar in AA (19.31 μmol/L), gangrenous/perforated cases (23.10 μmol/L), and non-appendicitis patients (17.27 μmol/L). At a cutoff of 10 μmol/L, sensitivity was 63% and specificity 33%; at 20 μmol/L, sensitivity dropped to 40% with specificity 63%. Limitations to this study included its small sample size and lack of pediatric data. The authors concluded that urinary 5-HIAA cannot reliably differentiate appendicitis from other abdominal pathologies, contradicting earlier studies reporting high accuracy (Table 1) (20).

Acharya et al. (21) conducted a systematic review and cost-benefit analysis of 62 studies (2000–2015) evaluating biomarkers for AA. Urinary 5-HIAA demonstrated high specificity (86%) and moderate sensitivity (72%) with an AUC of 0.88, outperforming traditional markers like WBC (AUC: 0.75) and CRP (AUC: 0.80) but requiring longer processing time (240 h) and higher cost (£21/test). IL-6 and procalcitonin showed superior diagnostic accuracy but were limited by cost and availability. The study emphasized trade-offs between diagnostic performance and practicality, concluding that no single biomarker is ideal for standalone diagnosis. Surgeons prioritized sensitivity (ranked #1) over cost, favoring combinations like WBC/CRP for clinical utility (Table 1) (21).

Ilkhanizadeh et al. (22) evaluated spot urinary 5-HIAA as a diagnostic biomarker for AA in 166 patients with acute abdominal pain, comparing results to 40 healthy controls. Using HPLC analysis, they found significantly elevated 5-HIAA levels in appendicitis patients (42.76 ± 2.26 μmol/L) vs. controls (*p* < 0.001). At a cutoff of 20 μmol/L, the test demonstrated exceptional diagnostic performance in clinically suspected cases (sensitivity: 98%, specificity: 100%, PPV: 100%, NPV: 93%). Notably, gastroenteritis patients showed similar 5-HIAA elevation (43.05 ± 2.7 μmol/L), representing a key limitation. The authors concluded that urinary 5-HIAA reliably confirms appendicitis when elevated and effectively rules out disease when normal, potentially reducing unnecessary surgeries (Table 1) (22).

Mentes et al. (23) conducted an experimental study using a rabbit model to evaluate urinary 5-HIAA (U-5-HIAA) in AA. They found significantly elevated U-5-HIAA levels (5.6 ± 0.1 mg/g creatinine) in early appendicitis (12-h ligation group) compared to controls (3.5 ± 0.6 mg/g creatinine, *p* = 0.003). At a cutoff of 4.15 mg/g creatinine, U-5-HIAA demonstrated 85% sensitivity and 64% specificity (AUC = 0.805) for diagnosing early appendicitis. Notably, levels decreased in later-stage/gangrenous cases. The study concluded that spot U-5-HIAA measurement may aid early appendicitis diagnosis but has limited utility in advanced cases (Table 1) (23).

Xu et al. (24) developed a novel HPLC method using poly(bromophenol blue)-modified electrodes to simultaneously measure urinary 5-HIAA and 5-HT. In 15 appendicitis patients, they found 5-HIAA levels were four-fold higher than controls (27.36 ± 3.24 vs. 6.73 ± 1.15 μmol/L, *p* < 0.01), while 5-HT showed no significant difference. The study proposed 20 μmol/L as a potential diagnostic cutoff for 5-HIAA but did not report sensitivity or specificity values. Limitations to this study include small sample size (*n* = 15 patients) precludes robust diagnostic accuracy assessment, critical performance metrics (sensitivity/specificity/AUC) are unreported, and the analytical focus limits direct clinical translation. This work provides important technical validation for 5-HIAA measurement methods, though its clinical conclusions are limited by small sample size and lack of complete diagnostic accuracy metrics (Table 1) (24).

### 3.2 Leucine-rich alpha-2 glycoprotein (LRG)

A study by Kakar et al. (25) evaluated the diagnostic utility of urinary LRG1 (u-LRG1) and serum LRG1 (s-LRG1) in pediatric acute appendicitis (PAA). The results showed that both u-LRG1 and s-LRG1 levels were significantly elevated in AA patients compared to controls (*p* < 0.001), with s-LRG1 demonstrating superior diagnostic accuracy (AUC = 0.95, sensitivity = 93.8%, specificity = 91.1%) compared to u-LRG1 (AUC = 0.70, sensitivity = 54.2%, and specificity = 83.9%). Notably, s-LRG1 effectively differentiated between complicated (AcA) and uncomplicated appendicitis (AuA; *p* = 0.001), while u-LRG1 did not (*p* = 0.089). The study also found that both biomarkers decreased significantly post-appendectomy, correlating with patient recovery. These findings suggest that s-LRG1 is a more reliable biomarker for AA diagnosis and severity assessment while u-LRG1, though less sensitive, remains a promising non-invasive tool. The study highlights the potential of integrating u-LRG1 with other urinary biomarkers to enhance diagnostic accuracy, particularly in resource-limited settings (Table 2) (25).

**Table 2:** Diagnostic performance of LRG in acute appendicitis.

**Study**	**Population**	**Biomarker**	**Cutoff**	**Sensitivity (%)**	**Specificity (%)**	**AUC**	**Notes**
Kakar, Berezovska (25)	153 patients (97 AA, 56 controls)	Serum LRG1	51.69 μg/ml (AA vs. control)	93.8	91.1	0.94 (0.91–0.99)	Superior diagnostic accuracy for AA
			84.06 μg/ml (AcA vs. AuA)	NR	NR	0.69 (0.59–0.80)	Differentiates AcA vs. AuA (*p* = 0.001)
		Urine LRG	0.18 μg/ml (AA vs. control)	54.2	83.9	0.70 (0.62–0.79)	No AcA/AuA differentiation (*p* = 0.089)
Yap, Fan (26)	34 pediatric patients (17 AA, 17 non-AA)	Urine LRG (creatinine-adjusted)	1.5 g/mol	17.65	100		Low sensitivity, high specificity
Arredondo Montero, Pérez Riveros (27)	712 participants (305 PAA, 407 controls)	Urinary LRG1 (creatinine-adjusted)	NR	NR	NR	Pooled MD: 0.89 g/mol (0.11–1.66) (creatinine-adjusted)	Meta-analysis: promising but needs standardization
Yap, Fan (28)	148 pediatric patients (42 AA)	Urinary LRG	NR	97.6	37.7%	0.82 (0.75–0.89)	AuB score < 0.15; Excellent for ruling out appendicitis
Gudjonsdottir, Roth (29)	173 pediatric patients (NA, UA, CA)	Urinary LRG1	0.10 μg/ml	80%	51%	AUC: 0.65 (0.55–0.75)	Significantly higher in complicated vs. uncomplicated appendicitis (*p* < 0.001)
Lontra, Savaris (39)	28 patients (14 AA, 14 non-AA)	Serum LRG1	NR	NR	NR	NR (Mann–Whitney test, *p* = 0.26)	No significant difference in plasma LRG1 levels between appendicitis (median 8.8 ng/ml) and non-appendicitis (median 11 ng/ml) groups
Salö et al. (30)	44 children (22 AA, 22 non-AA)	Urinary LRG (creatinine-adjusted)	≥0.036 g/mol	86	73	0.86 (0.79–0.99)	LRG higher in gangrenous/perforated vs. phlegmonous AA (*p* = 0.003)
Kentsis et al. (31)	49 children (24 AA)	Urinary LRG	NR	NR	NR	0.98 (0.96–1.0)	100 × higher in AA vs. controls and correlates with histologic severity
Kharbanda, Rai (32)	137 children (58 AA)	Urinary LRG (ELISA)	< 42 ng/ml (rule-out)	100 (91–100)	23 (15–34)	0.63 (0.52–0.73)	3 × higher in AA vs. controls and 81 × higher in perforated vs. non-perforated AA Inferior to WBC (AUC 0.82)
Mahalik, Bandyopadhyay (33)	41 children (3–16 years)	Urinary LRG	NR	NR	NR	0.586	No significant cutoff identified; poor diagnostic performance
Kentsis, Lin (35)	67 children (median age: 11 years)	Urinary LRG	NR	NR	NR	0.97	High diagnostic accuracy; correlates with disease severity. Limited specificity due to elevation in other inflammatory conditions (e.g., pyelonephritis)

AA, acute appendicitis; AcA, acute complicated appendicitis; AuA, acute uncomplicated appendicitis; NA, no appendicitis; UA, uncomplicated appendicitis; CA, complicated appendicitis; AuB, appendicitis urine biomarker score; PAA, pediatric acute appendicitis; MD, mean difference; NR, not reported; AUC, area under the curve.

Yap et al. (26) conducted a prospective, blinded, case-control study to evaluate the diagnostic utility of leucine-rich alpha-2-glycoprotein (LRG) in urine and saliva for identifying AA in pediatric patients. The study included 34 patients (17 with AA and 17 without) aged 4–16 years, with urine and saliva samples collected preoperatively. Urinary LRG levels were normalized against creatinine and analyzed using ELISA. The results showed that urinary LRG had a sensitivity of 17.65 and 100% specificity at a cutoff of 1.5 g/mol, correctly identifying only three AA cases. This performance was inferior to salivary LRG, which demonstrated higher diagnostic accuracy. The authors highlighted the challenges of urine collection in pediatric patients, including delays in obtaining samples and variability in urine concentration, which may have contributed to the limited utility of urinary LRG. Despite these limitations, the study underscores the potential of non-invasive biomarkers like LRG for diagnosing AA, particularly in pediatric populations. However, the low sensitivity of urinary LRG suggests it is not a reliable standalone diagnostic tool for AA, aligning with the broader need for further research to refine urinary biomarkers and improve their clinical applicability in appendicitis diagnosis. These findings contribute to the ongoing exploration of urinary biomarkers in AA, emphasizing the importance of integrating them with clinical and imaging findings to enhance diagnostic accuracy (Table 2) (26).

Arredondo Montero et al. (27) conducted a systematic review to evaluate the diagnostic performance of Leucine-Rich Alpha-2-Glycoprotein (LRG1) in PAA, focusing on its potential as a non-invasive biomarker. The review synthesized data from eight prospective studies involving 712 participants (305 PAA cases and 407 controls) and performed four meta-analyses to assess LRG1 levels in serum, saliva, and urine. The results demonstrated that LRG1 levels were significantly higher in PAA patients compared to controls, with pooled mean differences of 46.76 μg/ml in serum, 0.61 μg/ml in unadjusted urine, and 0.89 g/mol in creatinine-adjusted urine. These findings suggest that urinary LRG1, in particular, holds promise as a non-invasive diagnostic tool for PAA, with potential applications in reducing diagnostic errors and improving patient outcomes. However, the review highlighted significant heterogeneity among studies, particularly in serum LRG1 measurements, which may limit its clinical utility. The authors also noted that urinary LRG1 adjusted for creatinine showed greater diagnostic potential, though further research is needed to standardize measurement protocols and validate these findings in larger, more diverse populations. Additionally, the pilot study on salivary LRG1 yielded promising results, though its diagnostic accuracy requires confirmation in larger studies. This systematic review underscores the importance of integrating urinary biomarkers like LRG1 into the diagnostic framework for PAA, particularly in cases where imaging and clinical findings are inconclusive. By providing a non-invasive alternative to blood-based tests, LRG1 could enhance diagnostic accuracy, reduce patient stress and improve the overall management of pediatric appendicitis (Table 2) (27).

Yap et al. (28) conducted a prospective, observational cohort study to evaluate the diagnostic utility of urinary LRG in children with suspected AA. The study included 148 patients aged 4–16 years, of whom 42 were diagnosed with AA, including nine cases of perforated appendicitis. Urine LRG levels, normalized to creatinine, were measured using ELISA and combined with clinical variables (constant pain, right iliac fossa tenderness, and pain on percussion/coughing/hopping) to develop the appendicitis urine biomarker (AuB) score. The AuB score demonstrated a sensitivity of 97.6% and a negative predictive value (NPV) of 97.5% at a cutoff of < 0.15, outperforming the pediatric appendicitis score (PAS) in ruling out AA. Urinary LRG alone showed moderate diagnostic performance, with an area under the ROC curve (AUC) of 0.80, comparable to traditional markers like white blood cell count (WBC) and C-reactive protein (CRP). The authors highlighted the non-invasive nature of urinary LRG as a significant advantage in pediatric practice, potentially reducing the need for imaging and hospitalization in low-risk patients. However, they emphasized the need for further validation in larger, multicenter studies to confirm its clinical utility. This study underscores the potential of urinary biomarkers, particularly LRG, as adjuncts in the diagnostic evaluation of pediatric AA, offering a promising alternative to invasive diagnostic methods (Table 2) (28).

Gudjonsdottir et al. (29) investigated the diagnostic potential of leucine-rich alpha-2 glycoprotein 1 (LRG1) as a urinary biomarker in pediatric patients with suspected appendicitis. In a prospective study of 173 children under 15 years of age, blood and urine samples were collected during clinical evaluation, and LRG1 concentrations were analyzed. The study found no significant differences in serum LRG1 levels among patients with no appendicitis (NA), uncomplicated appendicitis (UA) and complicated appendicitis (CA). However, urine LRG1 levels were significantly lower in children with UA compared to those with NA and CA (*p* < 0.001), suggesting that urine LRG1 may have potential as a non-invasive biomarker for distinguishing between these conditions. This study highlights the potential utility of urine LRG1 in differentiating uncomplicated from complicated appendicitis in pediatric patients. The results contribute to the growing body of evidence supporting the role of urinary biomarkers in the diagnosis and management of AA (Table 2) (29).

Salö et al. (30) evaluated the performance of leucine-rich alpha-2 glycoprotein (LRG) as a urinary biomarker for AA in children. The study found that LRG levels were significantly elevated in children with AA, particularly in severe cases such as gangrenous or perforated appendicitis. When combined with the pediatric appendicitis score (PAS), LRG demonstrated high sensitivity and specificity, making it a promising biomarker for pediatric AA. Additionally, Zhao et al. (40) identified a 10-protein panel in urine that could distinguish AA from other acute abdominal conditions with an accuracy of 83.6%, suggesting the potential of urinary proteomics in AA diagnosis. These findings contribute to the growing evidence on urinary biomarkers for appendicitis, highlighting their potential in improving diagnostic accuracy, particularly when combined with clinical scoring systems (Table 2) (30).

Kentsis et al. (31) conducted a prospective study evaluating urinary leucine-rich alpha-2-glycoprotein (LRG) in 49 children with suspected appendicitis (24 confirmed cases). Using mass spectrometry (SIM MS), they found LRG levels were >100-fold higher in appendicitis patients vs. controls (AUC = 0.98, 95% CI 0.96–1.0), outperforming standard markers like CRP (AUC = 0.76) and neutrophil count (AUC = 0.73). However, commercial ELISA measurements showed interference effects, reducing diagnostic accuracy (AUC = 0.80). LRG levels correlated with histologic severity, with severe cases showing significantly higher concentrations than moderate appendicitis (*p* = 0.06). The study highlights LRG's potential as a highly sensitive biomarker but emphasizes the need for improved assay development to overcome technical limitations (Table 2) (31).

Kharbanda et al. (32) prospectively evaluated urinary LRG in 137 children with suspected appendicitis (58 confirmed cases). While median urinary LRG levels were significantly higher in appendicitis patients (683.5 ng/ml) vs. controls (225.2 ng/ml, *p* = 0.008), the biomarker showed limited diagnostic accuracy (AUC = 0.63, 95% CI: 0.52–0.73). Notably, urinary LRG demonstrated excellent discrimination for perforated appendicitis (20,576.8 ng/ml vs. non-perforated 252.7 ng/ml, *p* < 0.001). At a cutoff of < 42 ng/ml, urinary LRG achieved 100% sensitivity and 23% specificity for ruling out appendicitis, though this performance was inferior to WBC counts. The study highlights urinary LRG's potential as a “rule-out” marker and its strong association with disease severity, while noting its limited standalone diagnostic value (Table 2) (32).

The study by Mahalik et al. (33) evaluated the diagnostic accuracy of urinary Leucine-rich α-2-glycoprotein (LRG) as a biomarker for pediatric appendicitis in an Indian cohort. The results demonstrated that LRG had poor diagnostic performance, with an AUC of 0.586 (95% CI: 0.407–0.766), and no specific cutoff value could be identified to differentiate appendicitis from mesenteric lymphadenitis. In contrast, the pediatric appendicitis score (PAS) showed better diagnostic accuracy (AUC: 0.821), with a cutoff of 6.5 yielding 80% sensitivity and 76.2% specificity. The study concluded that LRG, when measured using a commercial ELISA kit, was not a reliable standalone biomarker and emphasized the superiority of PAS for clinical decision-making. The authors suggest that methodological refinements, such as mass spectrometry, may improve LRG's utility in future studies. These findings align with previous research highlighting the challenges of using LRG as a diagnostic tool, particularly due to its non-specific elevation in inflammatory conditions (Table 2) (33).

The study by Gurushankari et al. (34) reviewed the diagnostic utility of urinary Leucine-rich alpha-2-glycoprotein (LRG) as a biomarker for AA, highlighting its variable performance across methodologies. While LRG demonstrated high accuracy (99%) when measured via mass spectrometry, its efficacy dropped significantly (80%) with conventional ELISA, underscoring methodological limitations. The biomarker's non-specific elevation in other inflammatory conditions (e.g., pyelonephritis) further limits its standalone diagnostic value. The authors noted that LRG levels rise earlier than neutrophils, suggesting potential for early detection, but emphasized the need for standardized assays to improve reliability. This study aligns with prior research indicating that LRG's diagnostic performance is context-dependent and may require complementary tools like clinical scoring systems for practical use (Table 2) (34).

The study by Kentsis et al. (35) utilized high-accuracy mass spectrometry to identify urinary Leucine-rich α-2-glycoprotein (LRG) as a highly sensitive and specific biomarker for acute pediatric appendicitis. In a prospective cohort of 67 children, LRG demonstrated exceptional diagnostic performance, with an AUC of 0.97 (95% CI: 0.93–1.0), outperforming other biomarkers like S100-A8 and α-1-acid glycoprotein 1. The study highlighted LRG's enrichment in diseased appendices and its correlation with disease severity, suggesting its role in local neutrophilic inflammation. Notably, LRG was detectable even in early-stage appendicitis, including cases with normal imaging findings. However, its elevation in conditions like pyelonephritis indicates limited specificity for appendicitis alone. The authors proposed that LRG's diagnostic utility could be enhanced through immunoassays, though mass spectrometry remains the gold standard for detection. This study underscores LRG's potential as a non-invasive biomarker but emphasizes the need for further validation in diverse clinical settings (Table 2) (35).

## 4 Discussion

The evaluation of urinary biomarkers for AA reveals distinct diagnostic profiles for 5-HIAA and LRG, each with unique strengths and limitations. While 5-HIAA's role in perforation detection (13) is mechanistically intriguing its overall discriminative capacity for AA remains modest (AUC ~ 0.64) and offers little incremental value over CRP/WBC in uncomplicated cases (16, 18). Clinical adoption is further hampered by barriers, particularly the impracticality of enforcing dietary restrictions in emergency settings. LRG emerges as the more viable option, though our analysis exposes a critical paradox: the creatinine-adjusted AuB score's impressive NPV (97.6%) (28) contrasts starkly with standalone urinary LRG's poor sensitivity (17.7%) (26). This discrepancy suggests that LRG's value lies entirely in its integration with clinical scoring systems, not as an independent biomarker. While 5-HIAA demonstrates biological plausibility for perforation detection, its clinical utility is significantly constrained by the requirement for stringent dietary controls. The need for 24-h dietary restrictions on serotonin-rich foods is impractical in emergency settings, where rapid decisions are critical. The apparent diagnostic superiority of LRG when creatinine-adjusted warrants critical examination, as this methodological approach may obscure its limited discriminative capacity in unadjusted measurements. This normalization may artificially inflate performance metrics. Calprotectin's diagnostic delay underscores the need for rapid-turnaround biomarkers like LRG in pediatric settings, particularly for timely identification of complicated cases (36).

Notably, Di Mitri et al. (37) underscore the role of radical surgery and targeted antibiotics in reducing postoperative complications, even amid high rates of resistance to traditional therapies like amoxicillin-clavulanate. These clinical challenges underscore the need for reliable biomarkers (e.g., LRG, 5-HIAA) to guide decisions, particularly in ambiguous presentations. A study conducted by Naji et al. (38) highlighted the importance of microbiological culture and sensitivity testing in guiding antibiotic selection for pediatric appendicitis. The predominance of *Escherichia coli* (85%) and its susceptibility profile support using amoxicillin/clavulanic acid empirically for perioperative prophylaxis, aligning with local protocols. However, the detection of resistant pathogens (27% sensitivity to cefazolin, for example) underscores the need for tailored regimens based on culture results, particularly in cases of perforation or gangrenous appendicitis where postoperative complications are more likely. These data reinforce that urine or tissue cultures, when available, should inform antibiotic stewardship to optimize efficacy and minimize resistance (38).

The QUADAS-2 evaluation revealed significant heterogeneity in methodological rigor across studies. While Mohammadi Tofigh et al. (13) and Kentsis et al. (35) demonstrated low overall bias (prospective designs, standardized assays, and histopathology confirmation), others like Jangjoo et al. (17) and Hernandez et al. (20) were high-risk due to selection bias (exclusion of conservatively managed cases) and inconsistent timing of biomarker sampling. Notably, meta-analyses (15, 27) highlighted spectrum bias from variable control groups (healthy vs. abdominal pain patients), undermining generalizability. Urinary LRG performed optimally in pediatric studies when integrated with clinical scores (e.g., AuB), but standalone sensitivity remained poor. Standardization of assays (e.g., mass spectrometry over ELISA) and prospective validation in diverse cohorts are critical to mitigate bias and enhance translational utility (Table 3).

**Table 3:** QUADAS-2 evaluating the risk of bias.

**Study**	**Patient selection**	**Index test**	**Reference standard**	**Flow and timing**	**Overall bias**
Haji Maghsoudi, Soltanian (12)	High risk: convenience sampling (surgical candidates only). No healthy controls included	Low risk: ELISA for 5-HIAA with standardized acidification/storage	Low risk: intraoperative/histopathologic confirmation for all cases	Unclear risk: timing of urine sampling relative to symptoms not detailed	Moderate risk (selection bias dominates)
Mohammadi Tofigh, Samsami (13)	Low risk: prospective design, included perforated and non-perforated cases	Low risk: ELISA with acidified storage, pre-surgery sampling	Low risk: histopathologic confirmation + surgical findings	Low risk: samples collected preoperatively with standardized timing	Low risk (balanced design)
Khirallah and Abdel Ghafar (14)	Low risk: randomized control trial, excluded confounding factors (e.g., drugs/foods)	Low risk: ELISA with creatinine adjustment, blinded analysis	Low risk: histopathologic confirmation	Low risk: preoperative sampling, clear follow-up for readmissions	Low risk (rigorous methodology)
Arredondo Montero, Bueso Asfura (15)	Low risk (6/12): prospective recruitment in emergency settings with clear inclusion criteria. High risk (1/12): excluded conservatively managed patients; potential spectrum bias. Unclear (5/12): insufficient details on recruitment methods	Low (9/12): pre-specified 5-HIAA cut-offs or blinded interpretation in most studies. Unclear (3/12): lack of details on test execution or thresholds	Low risk (11/12): histopathological confirmation of appendicitis used in most studies. High risk (1/12): reliance on clinical diagnosis without histopathology	Unclear (12/12): no study explicitly described time intervals between index test and reference standard	Moderate risk: heterogeneity in control groups (healthy vs. abdominal pain patients) and inconsistent reporting of methodological details
Bosak Versic, Glavan (16)	High risk: convenience sampling (surgical candidates only). No healthy controls included in primary analysis	Low risk: blinded 5-HIAA measurement via HPLC, standardized protocol	Low risk: intraoperative/histopathologic confirmation for all cases	Unclear risk: timing of urine sampling relative to symptoms not detailed	Moderate risk (selection bias dominates)
Jangjoo, Varasteh (17)	High risk: excluded conservatively managed patients; potential spectrum bias	Low risk: ELISA for 5-HIAA, though method may differ from HPLC	Low risk: histopathologic confirmation for operated cases	High risk: delayed sampling (timing post-admission unclear)	High risk (selection/timing bias)
Rao, Wilson (18)	Low risk: consecutive recruitment, included both surgical and non-surgical cases	Low risk: ELISA with acidified storage, though method variance exists	Low risk: histopathology or CT for diagnosis	Low risk: samples collected within 24h of admission	Low risk (balanced design)
Bolandparvaz, Vasei (19)	High risk: healthy controls used for comparison (not clinically relevant)	Low risk: HPLC with rigorous protocol	Low risk: histopathologic confirmation	Unclear risk: sampling time post-symptom onset not reported	Moderate risk (selection bias)
Hernandez, Jain (20)	High risk: excluded conservatively managed patients; potential spectrum bias (only surgical cases analyzed)	High risk: urinary 5-HIAA cutoff values varied; no blinding mentioned	Low risk: histopathology confirmed appendicitis in surgical cases	High risk: discharged patients not followed; potential missed appendicitis cases	High risk
Acharya, Markar (21)	Low risk: included consecutive patients with suspected appendicitis; broad inclusion criteria	Unclear risk: biomarker thresholds not always predefined; blinding not explicitly stated	Low risk: histopathology used as gold standard for all surgical cases	Low risk: minimal attrition; clear follow-up for all included patients	Low risk
Ilkhanizadeh, Owji (22)	High risk: excluded conservatively managed patients (only surgical cases analyzed); healthy controls used for comparison, which may not reflect real-world diagnostic challenges	Unclear risk: 5-HIAA cutoff (20 μmol/L) was predefined, but blinding of assessors to histopathology results not mentioned	Low risk: histopathology used as gold standard for appendicitis cases	High risk: discharged patients (*n* = 7) not followed long-term; potential missed appendicitis cases	High risk
Mentes, Eryilmaz (23)	High risk: animal model (rabbits); may not fully replicate human AA pathology	Low risk: HPLC used for 5-HIAA measurement; standardized protocols	High risk: animal model limitations; histopathology may not mirror human disease	Unclear risk: timing of urine collection post-ligation not fully detailed	High risk: animal study with translational limitations to human diagnosis
Xu, Zhang (24)	Low risk: included confirmed AA patients and controls; no exclusion criteria described	Low risk: HPLC-ED method validated with clear protocols; blinded analysis	Low risk: histopathology (gold standard) used for AA diagnosis	Low risk: all samples processed uniformly; no timing issues reported	Low risk: balanced design with clear methodology
Kakar, Berezovska (25)	Low risk: prospective cohort with clear inclusion/exclusion criteria; balanced groups	Low risk: s-LRG1/u-LRG1 measured via ELISA; predefined cut-offs	Low risk: s-LRG1/u-LRG1 measured via ELISA; predefined cut-offs	Kakar et al. (25)	Low risk: prospective cohort with clear inclusion/exclusion criteria; balanced groups
Yap, Fan (26)	Low risk: prospective recruitment of children with suspected AA; clear exclusion criteria (e.g., chronic conditions)	Low risk: salivary LRG measured using standardized ELISA; blinded analysis	High risk: non-AA group confirmed via clinical follow-up (no histopathology)	Low risk: uniform sample collection and processing; no timing issues reported	Moderate risk: non-AA group lacked histopathology confirmation
Arredondo Montero, Pérez Riveros (27)	Moderate risk: most studies prospectively recruited children with suspected appendicitis, but control groups varied (e.g., healthy vs. non-appendicitis abdominal pain). Some studies lacked histopathology confirmation for controls, introducing potential spectrum bias	Low risk: LRG1 levels were measured using standardized ELISA or mass spectrometry in most studies, with predefined cut-offs. Blinding was often reported, reducing test interpretation bias	Moderate risk: while most studies used histopathology to confirm appendicitis, some relied on intraoperative findings or clinical follow-up for controls. Heterogeneity in reference standards (e.g., culture results vs. histopathology) may affect accuracy	Low risk: uniform protocols for sample collection and timing were generally followed. All studies obtained samples preoperatively, minimizing misclassification due to disease progression	Moderate risk: despite robust methods in individual studies, variability in control group definitions and reference standards introduced moderate bias. High heterogeneity in serum LRG1 meta-analysis further supports this
Yap, Fan (28)	High risk: excluded conservatively managed patients; potential spectrum bias as only surgically evaluated cases were included	Low risk: AuB score was clearly defined and pre-specified; urine LRG analysis was blinded to reference standard	Low risk: histopathology (for appendicitis) and clinical follow-up (for non-appendicitis) were appropriate and blinded to index test	Unclear risk: timing of urine sample collection relative to clinical presentation was not detailed; potential variability in sample handling	High risk in patient selection due to exclusion of non-surgical cases; other domains were low/unclear
Gudjonsdottir, Roth (29)	High risk: excluded conservatively managed patients; potential spectrum bias as only surgically evaluated cases were included. High appendicitis prevalence (77%) suggests a selective cohort	Low risk: serum and urine LRG1 were analyzed using standardized ELISA methods, and results were interpreted without knowledge of the reference standard	Low risk: appendicitis diagnosis and severity were confirmed via histopathology and intraoperative findings, which are considered gold standards. Non-appendicitis cases were followed clinically	Unclear risk: timing of sample collection relative to symptom onset was not detailed. Missing data for 27 patients (excluded) may introduce bias	High risk in patient selection due to exclusion of non-surgical cases and high appendicitis prevalence. Other domains were low/unclear
Salö, Roth (30)	High risk: excluded conservatively managed patients; potential spectrum bias as only surgically evaluated cases were included. Non-random inclusion (only when specific authors were on call) may introduce selection bias	Low risk: urinary biomarkers (LRG, calprotectin, IL-6, substance P) were analyzed using standardized ELISA methods, blinded to reference standard results	Low risk: appendicitis diagnosis confirmed via histopathology (gold standard). Non-appendicitis cases were followed clinically to confirm alternative diagnoses	Unclear risk: timing of urine sample collection relative to symptom onset not detailed. Exclusion of 23 appendicitis and 93 non-appendicitis patients due to non-inclusion by on-call authors may affect generalizability	High risk in patient selection due to non-random inclusion and exclusion of non-surgical cases. Other domains were low/unclear
Kentsis, Ahmed (31)	High risk: excluded conservatively managed patients; potential spectrum bias as only surgically evaluated cases were included. Convenience sampling may introduce selection bias	Low risk: urine LRG was analyzed using both ELISA and SIM mass spectrometry (MS), with results interpreted blinded to the reference standard. MS methods were robust but ELISA showed interference effects	Low risk: appendicitis diagnosis was confirmed via histopathology (gold standard). Non-appendicitis cases were followed via telephone to confirm alternative diagnoses	Unclear risk: timing of urine sample collection relative to symptom onset was not detailed. Small sample size (*n* = 49) may limit generalizability	High risk in patient selection due to convenience sampling and exclusion of non-surgical cases. Index test (ELISA) had limitations due to interference, while MS method was robust
Kharbanda, Rai (32)	High risk: excluded patients with prior abdominal surgery or chronic illnesses; potential spectrum bias as the study population may not represent all children with suspected appendicitis	Low risk: biomarker assays (calprotectin and LRG) were performed using standardized ELISA protocols, and lab personnel were blinded to the diagnosis	Unclear risk: final diagnosis was based on histopathology (for surgery) or follow-up (for non-surgery), but no details on blinded adjudication or consistency in reference standard application	Low risk: all enrolled patients were accounted for in the analysis, and biomarker testing was performed uniformly. Minimal delays in sample processing were addressed	High risk due to concerns in patient selection (spectrum bias) and unclear reference standard application, despite low risk in other domains
Mahalik, Bandyopadhyay (33)	High risk: excluded patients with chronic illnesses or prior abdominal surgery; potential spectrum bias as the study population may not represent all children with suspected appendicitis. Additionally, 20 patients were excluded due to incomplete data, which could introduce selection bias	Low risk: urinary LRG was measured using standardized ELISA protocols, and the investigator was blinded to patient information. However, the assay method (ELISA) may have lower accuracy compared to mass spectrometry, as noted in the discussion	Unclear risk: final diagnosis was based on clinical judgment, radiology (US/CT), and histopathology (for surgical cases). However, no details were provided on whether reference standard adjudicators were blinded to index test results or how conservatively managed cases were confirmed	Low risk: all enrolled patients were accounted for in the analysis, and urine samples were processed uniformly. Follow-up was conducted for conservatively managed cases, minimizing attrition bias	High risk due to concerns in patient selection (spectrum bias and exclusions) and unclear reference standard application, despite low risk in other domains
Gurushankari, Sureshkumar (34)	High risk: the review article synthesizes data from multiple studies with varying inclusion/exclusion criteria, some of which may exclude atypical presentations or conservatively managed cases, leading to potential spectrum bias. No uniform patient selection protocol across studies	Unclear risk: the article discusses multiple biomarkers (e.g., LRG, calprotectin, MPV) and scoring systems (e.g., Alvarado, PAS) but does not specify whether index tests were interpreted without knowledge of reference standards in primary studies. Variability in assay methods (e.g., ELISA vs. mass spectrometry for LRG) may affect accuracy	Unclear risk: the reference standard for appendicitis diagnosis (e.g., histopathology, imaging, clinical follow-up) varies across studies. Some studies may rely on imaging (CT/USG) without surgical confirmation, while others use histopathology, introducing inconsistency. No mention of blinding in primary studies	Unclear risk: the review does not provide details on whether all patients in primary studies received the same reference standard or if there were delays between index tests and reference standards. Attrition bias is not addressed	High risk due to heterogeneous patient selection, variability in index tests and reference standards, and lack of clarity on blinding and timing in primary studies
Kentsis, Lin (35)	Low risk: patients were prospectively enrolled based on clinical suspicion of appendicitis, including surgical consultation or imaging. Exclusions (e.g., pre-existing renal/autoimmune diseases) were justified to reduce confounding. The study included histologically confirmed cases and controls with similar demographics	Low risk: high-accuracy mass spectrometry (LC-MS/MS) was used for biomarker discovery and validation, with rigorous protocols for urine processing and analysis. Measurements were blinded to the final diagnosis, minimizing interpretation bias	Low risk: appendicitis diagnosis was confirmed by histopathology (gold standard) reviewed by a clinical pathologist with independent blinded confirmation. Non-appendicitis cases were confirmed via follow-up. This ensures high diagnostic accuracy	Low risk: all enrolled patients were accounted for, with no attrition bias. Urine samples were processed uniformly within 6 h of collection. Timing between index tests and reference standard was consistent (histopathology post-surgery or follow-up)	Low risk due to prospective design, blinded testing, gold-standard reference, and minimal attrition

### 4.1 Comparative analysis of urinary biomarkers

The diagnostic utility of 5-HIAA in AA remains contentious, with studies reporting conflicting results. Meta-analytic data from Arredondo Montero et al. (15) confirm that while 5-HIAA levels are elevated in AA (mean difference: 23.30 μmol/L, *p* < 0.001), its modest sensitivity (68.6%) and specificity (82%) reflect substantial heterogeneity (*I*^2^ = 97%). This variability stems from differences in control group composition (e.g., healthy controls vs. patients with non-AA abdominal pain, as highlighted by the authors), methodological differences (e.g., HPLC vs. ELISA) and uncontrolled confounders, particularly dietary intake of serotonin-rich foods (bananas, tomatoes, and nuts) and medications (aminosalicylates) (12, 28). While Mohammadi Tofigh et al. (13) reported 5-HIAA's utility in identifying perforated appendicitis (sensitivity: 82%, specificity: 62%), other studies found no significant differences between AA and non-AA groups Haji Maghsoudi et al. (12), Bosak Versic et al. (16). Notably, Khirallah and Abdel Ghafar (14) observed high sensitivity (91.8%) in pediatric AA when combined with the pediatric appendicitis score (PAS; AUC: 0.958), but Bosak Versic et al. (16) found no discriminative power (AUC: 0.55) in children, underscoring the impact of dietary controls and assay methods. The requirement for pre-test dietary restrictions (15, 16) and the biomarker's inability to distinguish between complicated/uncomplicated AA (15, 18) limit its clinical feasibility. Hernandez et al. (20) further noted poor diagnostic accuracy in adults (sensitivity: 40%−63%), while Ilkhanizadeh et al. (22) reported exceptional performance (sensitivity: 98%), this discrepancy likely attributable to variations in control groups (e.g., healthy vs. gastroenteritis patients).

By contrast, LRG demonstrates superior clinical feasibility as it remains unaffected by dietary factors, though its diagnostic utility varies significantly based on sample type (serum vs. urine), measurement technique (ELISA vs. mass spectrometry), and normalization (creatinine adjustment). Kakar et al. (25) demonstrated serum LRG's high accuracy (AUC: 0.95, sensitivity: 93.8%) for AA diagnosis and severity stratification, while urinary LRG (u-LRG1) underperformed (AUC: 0.70). This aligns with Lontra et al. (39), who found no significant difference in plasma LRG between AA and non-AA cases using ELISA, highlighting assay-specific limitations. Creatinine-adjusted urinary LRG shows promise in pediatric populations. The AuB score (combining u-LRG with clinical variables) achieved an NPV of 97.6% (28) outperforming PAS for ruling out AA. However, standalone u-LRG sensitivity is poor [17.7% (26)], and its performance varies by methodology. AuB algorithm's diagnostic power derives primarily from its incorporated clinical variables (e.g., RLQ tenderness, pain on coughing), not LRG alone. This discrepancy underscores that urinary LRG's utility in AA diagnosis is contingent on contextual clinical data, rather than functioning as an independent biomarker, this highlights the potential of multimodal diagnostics but does not isolate LRG's independent contribution. Mass spectrometry showed exceptional accuracy [AUC: 0.98 (31)] and correlation with histologic severity, whereas ELISA had limited utility [AUC: 0.63 (32)] due to interference effects (31, 34). LRG's elevation in other inflammatory conditions [e.g., pyelonephritis (34)] and inconsistent differentiation of uncomplicated/complicated AA (29, 33) underscore its role as an adjunct, not a standalone test.

### 4.2 Clinical implementation roadmap

While treatment strategies for pediatric appendicitis remain debated, urinary biomarkers may help guide both diagnosis and management decisions (1). Based on current evidence, we propose a tiered approach integrating urinary biomarkers with existing diagnostic algorithms. For initial triage in pediatric cases, the investigational creatinine-adjusted AuB score (cutoff < 0.15) combined with PAS (< 4) may help exclude appendicitis (NPV 97.6% in single-center studies) but requires prospective multicenter validation before clinical adoption. Until then, imaging remains the gold standard for equivocal cases. In equivocal cases (PAS 4–7), elevated urinary 5-HIAA (>15 mg/g creatinine) may help identify perforation risk, though dietary controls are advised. For adults, serum LRG (AUC: 0.95) shows promise but requires venipuncture.

Future work should validate cutoffs in diverse cohorts and develop POC platforms. These biomarkers should augment, not replace, clinical judgment and imaging (1), particularly given the importance of accurate severity assessment for antibiotic stewardship in the antimicrobial resistance era (1).

## 5 Limitations and future directions

Current evidence for 5-HIAA and LRG as urinary biomarkers in AA is limited by methodological inconsistencies, including variable assay techniques (ELISA vs. HPLC for 5-HIAA; creatinine-adjusted vs. unadjusted LRG) and confounding factors like diet (affecting 5-HIAA) and non-appendiceal inflammation (elevating LRG). The QUADAS-2 assessment further highlights key limitations: selection bias from excluding non-surgical cases, heterogeneous reference standards, and variability in sample timing and blinding. Additional constraints include small sample sizes, lack of standardized cutoffs, and unaddressed dietary/medication confounders. While some studies employed rigorous designs, these inconsistencies underscore the need for standardized protocols and prospective validation to establish reliable clinical utility. While 5-HIAA's specificity (~82%) appears robust, its low AUC in meta-analyses (~0.64) and failure to outperform routine biomarkers (CRP/WBC) in some studies suggest its utility may be restricted to perforation risk stratification rather than primary diagnosis. 5-HIAA may aid perforation risk assessment in controlled settings (13, 23) but is impractical for emergency use due to dietary confounders. LRG is best integrated with clinical scores (e.g., PAS) for pediatric triage (28, 30). Serum LRG is superior for diagnosis, but urinary LRG (creatinine-adjusted) offers non-invasive rule-out potential. Point-of-care urinary assays may enhance feasibility in emergency settings, particularly for pediatric cases where non-invasive tools are prioritized. Biomarker assays are often unavailable in Emergency Departments, necessitating rapid POC tests for clinical utility. Discrepancies in LRG performance [ELISA vs. MS; (31, 35)] and 5-HIAA's variability [HPLC vs. ELISA; (15, 24)] call for standardized protocols (e.g., creatinine adjustment for LRG, dietary controls for 5-HIAA) and larger multicenter studies to validate cutoff values and assess reproducibility across diverse settings. Future clinical adoption of these biomarkers hinges on standardized assays, cost-effectiveness analyses, integration into POC platforms, quantification of LRG's incremental diagnostic value beyond clinical factors alone. Our analysis included studies evaluating LRG both as a standalone biomarker and as part of composite scores (e.g., AuB), while this reflects real-world clinical integration, it risks conflating the performance of LRG itself with that of broader diagnostic algorithms. This limitation underscores the need for standardized reporting of biomarker contributions in multimodal tools. Urinary LRG, combined with clinical scores (e.g., PAS), may expedite triage in pediatric EDs, while 5-HIAA could aid perforation risk stratification in resource-limited settings.

## 6 Conclusion

In summary, 5-HIAA and LRG represent complementary but imperfect biomarkers for AA. 5-HIAA excels in detecting perforation but lacks consistency for general diagnosis, while LRG especially in urine, provides a practical, non-invasive adjunct but requires refinement to improve sensitivity. The proposed biomarker-assisted algorithms (e.g., AuB + PAS) remain investigational and must be validated in diverse, multicenter cohorts. Neither biomarker alone suffices for definitive diagnosis, but their combined use with clinical scoring systems may reduce diagnostic uncertainty, particularly in ambiguous cases. Future research should prioritize standardized assays, larger multicenter validations, and exploration of multimodal biomarker panels to optimize their clinical utility. Until then, these biomarkers remain promising adjuncts rather than replacements for existing diagnostic strategies.
